# Cases of Local Irritation, from the Presence of Extraneous Substances

**Published:** 1845-08-01

**Authors:** WM. Treat


### Cases of Local Irritation, from the Presence of Extraneous Substances.

Communicated for the Buffalo Medical Journal, by WM. TREAT, M. D.

Mr. Editor : — If the following cases should be deemed of sufficient interest to serve the purposes of your Journal, they are at your disposal.

*Foreign substance in the eye* : — R. N. age twenty-five, house carpenter, of good habits and constitution, was visited on the 4th Feb., 1844. I learned that on the 12th January while engaged in turkey-shooting, after a round of unsuccessful shots, it again became his turn, when, on the discharge of his rifle, a "*blur*" passed over his right eye, accompanied with no sensation of pain or other inconvenience, save this blur was sufficient to induce him to speak of it immediately to his companions, and call attention from the object of his sports, a successful shot and dead turkey. After some little delay he called upon a Physician in the vicinity, who, on examination with a microscope, diagnosed incision of the cornea, presumed to have been by the percussion cap; the agent was thought to have passed without the eye as it could not be seen, and for prognosis it was thought possible that the pupilary margin might become irregularly shaped. The remedies prescribed not preventing active inflammation, the patient was induced by friends to consult an itinerant oculist, then in the city, who resorted to mercurials; and the patient at this inclement season was in the habit of walking a mile to visit the 'Doctor,' while under profuse salivation. This course did not improve the original malady.

With this, the history, I found on examination chemosis, so that a large portion of the cornea was covered by the projecting conjunctiva, and the visible portion with injected vessels extending over it, and its structure marbled-opake. He was subjected to V. S., cathartics, restricted diet, and a darkened room, with ung. Belladonnæ to the brow. The inflammation was soon removed except a pink, sclerotic, marginal zone around the cornea.

14th February, a superficial ulcer appears upon the surface of the continuedly opake cornea, which was healed by the 4th of March.

26th March : Patient is severedly attacked with symptoms of internal opthalmia, which alternately were relieved and re-appeared, until the 20th May, when they finally disappeared, *with now marked diminution of the globe of the eye.* Pain complained of, much resembling that, as described, from dislocation of the lens. Traces of the pink zone around the eye continued.

Every few months the patient returned to me from his labor, complaining of great sensitiveness of the globe, orbital pain, and slight conjunctival inflammation, which were readily subdued.

On the 25th June, 1845, he had a return of these attacks, more serious than before, for which I resorted to V. S. ℥ xxii, nauseants, with low diet, &c., under which the symptoms subsided, and the patient so far recovered as to walk in procession of the 3d July.

4th July : There was a slight recurrence of symptoms. On the 5th I saw him. The cornea presented in its centre, a slightly transparent, pin-head like projection as though by pointing of an abscess. As vision was lost before my first visit, and had not returned, and there was now certainly no hope for it, and the globe was not sufficiently contracted to admit of the insertion of an artificial eye, I resolved to discharge the contents of the globe, believing an irritant, probably pus, to be within. A free incision was made through the cornea, and warm fomentations applied to the eye—no inflammation ensued.

8th July: On examination, the globe had not shrunk by reason of the opening. There was found presenting at the incision and where the apparent pointing appeared, i. e. at the centre of the cornea, a foreign substance which was removed by a pointed instrument and forceps, and proved to be *half of the rim or band of the percussion cap*, which was unchanged, chemically, though it had remained in the globe for 18 months.

The thought presents itself, how was effected the contraction of the globe to one half its natural size, when there was no external wound. The result in this case may assist in drawing practical deductions as to the propriety of extracting foreign bodies when known to be, and favorably located within the globe, notwithstanding the authority of Lawrence and others of broken cataract needles *disappearing* without injurious consequences. From the specific gravity of the body—the metalic cap—and the place where it presented, I infer that it must have been imbedded in the lens and thus upheld, with a projecting end to irritate the iris, and on the contraction of the globe, the cornea also, and thus cause the ulcerative absorption of the latter, and pointing, preparatory for its discharge.

While upon the subject of lodgment and retention of foreign bodies, permit me to relate the following, which, though presenting nothing new to the surgeon, may be of interest to him who reads for curiosity.

In 1836, a sailor presented himself with a tumor, situate in the space which may be best designated as the "web," between the index and median finger; this pricked and pained on pressure. For history, he related that about two years before, he was engaged in rowing a boat for escape from officers of the customs in the West Indies, when a shot struck his oar, splintering it, and injuring his hand. This was dressed after his captivity, and healed kindly, but ever after he had felt this pricking when at labor, *which had not been interrupted on account of it.* He now applied for relief. It was removed and proved on examination to be a piece of wood ¾ inch long and irregularly jagged, and perfectly inclosed in a cyst or sack, although it had thus been constantly subjected to motion in this, the hand of a sailor.

To this I add this recent case. Susan ———, a girl at service, who, during the accustomed house cleaning in May last, while engaged in window cleaning, by a rapid and violent downward motion thrust three fine cambric needles, which were sticking carelessly in the sash, transversely into the wrist. Two of them were readily removed as they projected from the surface; the third was imbedded and out of sight. From its position and the resistance which the carpal bones were hoped to have presented to this needle passing eye first, and the external puncture being perceptible, together with the fact that on motion of the hand or fingers, or by pressure over the spot, a pricking sensation was felt, and thus locating it, I was induced to make incision through the skin, though it proved to no purpose, and I could not feel justified to search farther, as the annular ligament lay immediately in its course. The arm was directed to be poulticed and kept in an elevated position ; much swelling of the hand and fingers ensued, and violent pain on attempt to move them. The swelling soon began to subside, and on the expiration of two weeks from the accident, the patient resumed her labor, experiencing only a slight pricking sensation when employed in the most *trying* labor, that of washing and wringing clothes. That the needle should not cause more inconvenience by reason of its peculiar location, renders this case a curious one, and the citation of it may serve to allay the anxiety of many who are often subjected to exposure, and often have needles thrust in the ball of the thumb when engaged in clothes washing.

That this needle will pursue the course of the many cases related of their tribe, and find its way to the surface, there is reason to believe, in accordance with that beautiful law of Nature which causes all foreign bodies to tend outwards, unless, as by rare exceptions, she rounds off angles, and clothes and guards them with cysts to restrain them from producing serious injury.

*Buffalo, July*, 1845.

